# Mutation of *Mycobacterium tuberculosis* and Implications for Using Whole-Genome Sequencing for Investigating Recent Tuberculosis Transmission

**DOI:** 10.3389/fpubh.2021.790544

**Published:** 2022-01-13

**Authors:** Kristin N. Nelson, Sarah Talarico, Shameer Poonja, Clinton J. McDaniel, Martin Cilnis, Alicia H. Chang, Kala Raz, Wendy S. Noboa, Lauren Cowan, Tambi Shaw, James Posey, Benjamin J. Silk

**Affiliations:** ^1^Rollins School of Public Health, Emory University, Atlanta, GA, United States; ^2^Division of Tuberculosis Elimination, National Center for HIV/AIDS (Human Immunodeficiency Virus/Acquired Immunodeficiency Syndrome), Viral Hepatitis, STD (Sexually Transmitted Diseases), and Tuberculosis (TB) Prevention, Centers for Disease Control and Prevention, Atlanta, GA, United States; ^3^Los Angeles County Department of Public Health, Los Angeles, CA, United States; ^4^TB Control Branch, California Department of Public Health, Richmond, CA, United States

**Keywords:** tuberculosis—epidemiology, prevention and control, genomic sequencing, tuberculosis transmission, public health practice

## Abstract

Tuberculosis (TB) control programs use whole-genome sequencing (WGS) of *Mycobacterium tuberculosis* (*Mtb*) for detecting and investigating TB case clusters. Existence of few genomic differences between *Mtb* isolates might indicate TB cases are the result of recent transmission. However, the variable and sometimes long duration of latent infection, combined with uncertainty in the *Mtb* mutation rate during latency, can complicate interpretation of WGS results. To estimate the association between infection duration and single nucleotide polymorphism (SNP) accumulation in the *Mtb* genome, we first analyzed pairwise SNP differences among TB cases from Los Angeles County, California, with strong epidemiologic links. We found that SNP distance alone was insufficient for concluding that cases are linked through recent transmission. Second, we describe a well-characterized cluster of TB cases in California to illustrate the role of genomic data in conclusions regarding recent transmission. Longer presumed latent periods were inconsistently associated with larger SNP differences. Our analyses suggest that WGS alone cannot be used to definitively determine that a case is attributable to recent transmission. Methods for integrating clinical, epidemiologic, and genomic data can guide conclusions regarding the likelihood of recent transmission, providing local public health practitioners with better tools for monitoring and investigating TB transmission.

## Introduction

Tuberculosis (TB) control programs use molecular characterization and surveillance of *Mycobacterium tuberculosis* (*Mtb*) for detecting TB case clusters, indicating or refuting possible epidemiologic links between patients, and defining an outbreak's magnitude and scope (1). Genotyping can also provide evidence that cases are attributable to recent TB transmission vs. reactivation of latent *Mtb* infection that was acquired during the remote past. Operationally, US TB programs often define recent transmission as an infection acquired during the previous 2 years (2), although no standard definition exists. Cases attributed to recent transmission are a priority for public health intervention because they can indicate existence of previously unrecognized infectious TB source cases or instances where contacts were not successfully identified or the evaluation for or treatment of TB infection was not completed. Genotyping based on a combination of spacer oligonucleotide typing (spoligotyping) and 24-locus mycobacterial interspersed repetitive unit–variable number tandem repeat (MIRU-VNTR) is performed for all culture-confirmed TB cases in the United States and is used to identify genotype-matched clusters (1). During 2012, the US Centers for Disease Control and Prevention's (CDC) Division of Tuberculosis Elimination (DTBE) began selectively performing whole-genome sequencing (WGS) for investigating clusters of genotype-matched TB cases for which recent transmission was suspected. As of 2018, CDC began prospectively performing WGS for all culture-confirmed TB cases in the United States, and clusters detected by genotyping now undergo rapid genomic characterization (3).

WGS can be used for identifying single nucleotide polymorphisms (SNPs) that distinguish *Mtb* isolates in a genotype-matched cluster. SNPs are mapped on a phylogenetic tree that diagrams the evolutionary direction of SNP accumulation among the isolates relative to a most recent common ancestor (MRCA), a hypothetical reference point from which all isolates in a given analysis are descended. The genetic distance between isolates is estimated by the number of SNPs between them, which provides a measure of their genetic relatedness. *Mtb* accumulates mutations slowly relative to other pathogens, but quickly enough that accumulation of SNPs can be observed over time. During TB disease, the *Mtb* genome has been estimated to acquire mutations at an average rate of 0.2–0.5 SNPs/genome/year, but this rate varies on the basis of characteristics of the infected person and the particular *Mtb* strain (4–7) (of note, the mutation rate and the substitution rate are distinct: the mutation rate reflects changes in the genome sequence between a parent and its offspring, or between isolates of cases that may be linked through transmission, whereas the substitution rate represents only those mutations that persist over time in the face of selection pressure). On the basis of DTBE's previous experiences using WGS and phylogenetic analysis for investigating recent transmission as well as numerous reports in the published literature (8–14), isolates that differ by >10 SNPs can usually be considered unlikely to be the result of transmission that occurred within the preceding 2 years (i.e., recent transmission). As SNP distances provide strong, although not definitive, evidence of recent transmission, local US TB programs commonly use a threshold approach, whereby cases with isolates that differ by ≤ 5 SNPs are prioritized for epidemiologic investigation.

**Figure 1:**
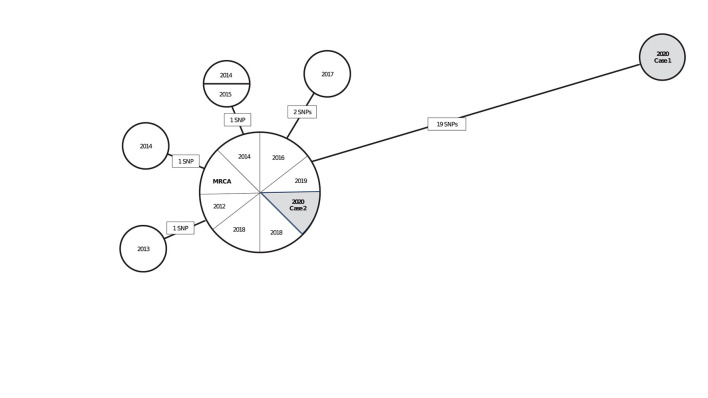
A hypothetical neighbor-joining tree (phylogenetic analysis) representing the genetic distances in single nucleotide polymorphisms (SNPs) among 15 isolates of *Mycobacterium tuberculosis* (*Mtb*) from culture-confirmed tuberculosis cases reported during 2010–2020^a^. ^a^Isolates are displayed as circles called *nodes*; isolates with the same genome sequence are displayed together in one node. Lines between nodes are labeled with the number of SNPs (mutations at a single position in the DNA sequence), and these lines are proportional in length to the number of SNPs. The most recent common ancestor (MRCA) is a hypothetical genome (not an actual isolate) from which all isolates in the phylogenetic analysis are descended. The MRCA serves as a reference point for examining the direction of genetic change. In this hypothetical scenario, a TB control program is using these phylogenetic analysis results to help determine if isolates from two patients reported during 2020 (shaded in gray) are likely attributable to recent transmission. If so, those cases are a priority for further investigation. Interpretation of these results, depending on if the *Mtb* mutation rate is assumed to be similar during latency or to be slower, is as follows: Case 1 is unlikely to be involved in recent transmission under either assumption because the patient's isolate is genetically distant to that of the patient in Case 2 and all other cases in the analysis (≥19 SNPs). This interpretation might change as new cases are reported and added to the phylogenetic analysis. Case 2 is more challenging to interpret: under an assumption that *Mtb* mutates at a similar rate during latent infection and disease, Case 2 is likely to be involved in recent transmission because the patient's isolate is genetically close to those of other cases in the analysis (0–2 SNPs). If Case 2 was attributable to reactivation after *Mtb* infection during the remote past, more SNPs can be expected. Under an assumption that *Mtb* mutates at a slower rate during latency than disease, Case 2 might be involved in recent transmission or attributable to reactivation after *Mtb* infection during the remote past because relatively few SNPs are expected to accumulate during latency. Other clinical and epidemiologic data are needed for making a determination.

Interpreting pairwise SNP differences by using a threshold approach should be done cautiously in light of TB's natural history, which is characterized by long and variable periods of latent infection. Concluding that a recent transmission event occurred between two persons can often depend on a key assumption regarding how quickly the *Mtb* genome accumulates mutations during latent infection. If we assume that this rate is similar to that during disease, we might expect *Mtb* sampled from a patient who had a long period of latent infection to have accumulated many SNPs relative to the isolate of the source patient from whom infection was acquired. If the mutation rate is slower during latent infection than during disease, we might expect little or no genomic change from the source patient's *Mtb* isolate to that of the secondary patient. In this scenario, interpretation of closely related isolates in the phylogenetic analysis is more uncertain, and sequencing results alone might not help resolve whether an infection was recently or remotely (>2 years prior) acquired. To illustrate, we provide a hypothetical but realistic example (Figure 1). In this hypothetical scenario, a TB control program is using phylogenetic analysis results to help determine if isolates from two patients reported during 2020 are likely attributable to recent transmission. Interpretation of results may depend on whether the *Mtb* mutation rate is assumed to be similar during latency as during disease or to be slower.

To date, extant literature regarding the *Mtb* mutation rate during latent infection has been limited and inconsistent (Appendix Table 1). The increasing availability of WGS data in the United States, combined with epidemiologic data from TB cluster investigations conducted by state and local TB programs, provide a rich data source for examining mutation rates during latent *Mtb* infection. First, we selected a series of well-characterized TB case clusters in which epidemiologic data provided strong support for linking source and secondary cases. We estimated the length of the secondary case's TB infection and examined the relationship between the duration of infection and accumulation of SNPs in the *Mtb* genome. Second, we describe a specific outbreak to illustrate the role and challenges of using genomic data for guiding conclusions regarding recent transmission in a programmatic setting. Our overarching goal is to help guide interpretation of SNP differences and improve TB programs' understanding of the genomic evidence required for reaching conclusions regarding recent TB transmission in such settings of low TB incidence as the United States.

## Materials and Methods

### Aggregated Cluster Data

For inclusion in our study, we considered all genotype-matched clusters of *Mtb* identified by molecular surveillance during 2015–2018 in Los Angeles County, California, that had ≥3 TB cases plus selected 2-case clusters for which transmission was suspected based on available epidemiologic information. A genotype match was defined on the basis of a combination of spoligotyping and 24-locus MIRU-VNTR results, allowing for a single-locus difference (15, 16). In addition, cases with epidemiologic links identified during cluster investigations were included. In California, health care providers are required by law to report TB cases to local health departments. The Los Angeles County TB Control program receives these case reports from local hospitals and providers and submits them to the California Department of Public Health, which compiles and submits them to CDC's National Tuberculosis Surveillance System (NTSS). Case reports include patient demographic information, medical and social risk factors for TB, and clinical, treatment, and outcome information (https://www.cdc.gov/tb/programs/rvct/rvct-form.pdf). A subset of all reported TB cases in Los Angeles County, including those belonging to genotype clusters with ≥3 cases plus select clusters of 2 cases, undergoes epidemiologic investigation for additional public health intervention. Data collected as part of epidemiologic investigations include symptom onset date and epidemiologic links to other TB patients.

On the basis of data collected for surveillance to infer infectious periods and epidemiologic links, presumed source patients were identified by local public health staff when possible. For each adult patient with pulmonary or laryngeal TB, we defined the infectious period start date, on the basis of published guidelines, as 3 months (or 4 weeks for patients with asymptomatic, smear-negative, and non-cavitary disease) before the earliest date that we could determine the patient had TB by using available surveillance and investigation data, including symptom onset date (17). The end of the infectious period was defined as the last date the patient had infectious TB or was able to transmit it to others (i.e., before placement in airborne isolation) on the basis of investigation data, if the local program had calculated this date; otherwise, the end date was defined as 2 weeks after treatment start date or the date of death, if applicable. Briefly, the strength of an epidemiologic link between a pair of patients was defined as (a) definite (e.g., a named contact to another patient during the TB infectious period); (b) probable (e.g., an association with the same location during or at the approximate time when one patient had infectious TB); or (c) possible (e.g., two patients lived or worked in the same neighborhood during the same general period (Appendix Table 2). We excluded pairs for which the WGS and phylogenetic analysis results were available before the source patient was designated, so that all source patient determinations were independent of WGS data. We compiled into a data set source and secondary patients identified by local public health staff, by using information regarding the estimated timing of epidemiologic links and clinical indicators of infectiousness. We refer to this data set as the *aggregated clusters*, because this group includes source–secondary case pairs from all the genotype-matched clusters.

### Single-Cluster Data

Separately, we also examined a single genotype-matched cluster of TB cases reported over the course of a 17-year period (2001–2017). Thirteen cases with this genotype were reported nationally, 10 of which were in California. An epidemiologic investigation of this California cluster identified 4 presumed source–secondary case pairs and a possible epidemiologic link for a fifth case. These cases were associated with a patient with infectious TB who had attended a California school during 2001.

### WGS and Phylogenetic Analysis

WGS was performed for *Mtb* isolates from the investigated cases, and whole-genome SNP comparison for all source–secondary case pairs for which quality sequence data were available and 10 isolates from California with matching genotype as part of the single-cluster investigation. Briefly, *Mtb* DNA were extracted using the Quick-DNA Fungal/Bacterial Kit (Zymo Research Corp., Irvine, CA, USA), and 1 ng was used to prepare sequencing libraries by using the NexteraXT Kit (Illumina, San Diego, CA, USA) according to the package insert. Libraries were sequenced on an Illumina MiSeq instrument to generate 250-bp paired-end reads. All analyses of WGS data were performed by using BioNumerics 7.6.3 (Applied Maths, Sint-Martens-Latem, Belgium). Reference guided assemblies were created using Bionumerics 7.6 Reference Mapper v 1.2.3 using *M. tuberculosis* strain H37Rv (NC00962.3) as the reference with the following settings for base calling: minimum total coverage = 3, minimum forward coverage = 1, minimum reverse coverage = 1, single base threshold = 0.75, double base threshold = 0.85, triple base threshold = 0.95, and gap threshold = 0.5. Average depth of coverage across the genome ranged from 42 to 222 with a median of 92. A list of high-quality, informative SNPs for each cluster was produced using the Strict SNP filtering (Closed SNP set) SNP analysis template for SNP filtering within the BioNumerics 7.6.3 software. For a SNP to be retained in the comparison, the base in all samples must have a total coverage of 5 reads, it must not be within 12 basepairs of another SNP, and it must not contain ambiguous bases, unreliable bases, or gaps. SNPs that are non-informative (identical in all samples) were also excluded. No genomic regions were specifically excluded from the analysis. For the single-cluster investigation, we constructed a phylogenetic tree by using the neighbor-joining method. Placement of the most recent common ancestor (MRCA) for isolates included in the phylogenetic tree was determined by rooting the tree with *Mtb* H37Rv as the outgroup.

### Statistical Analyses

**Figure 2:**
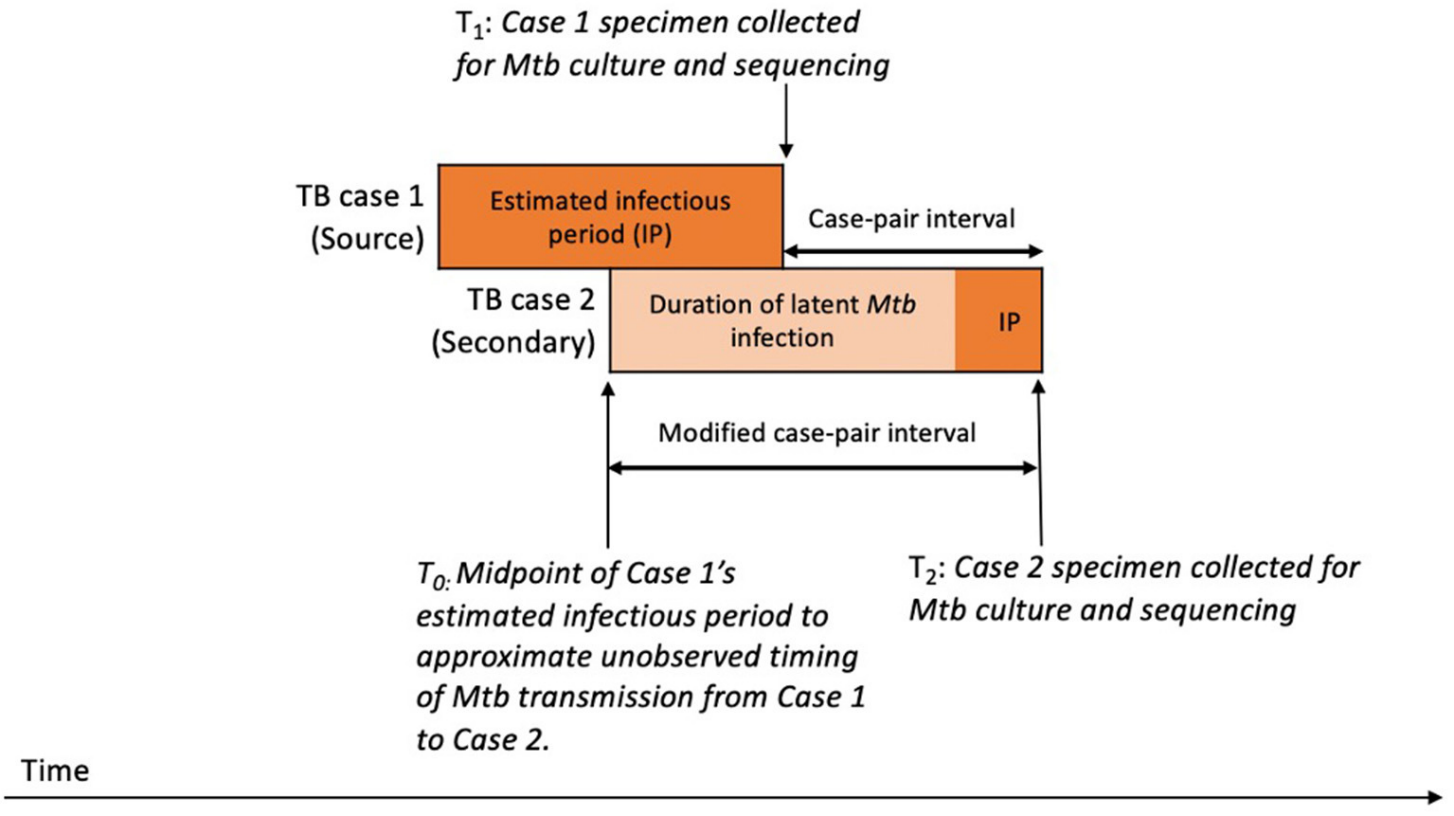
Schematic timeline of infection and transmission of *Mycobacterium tuberculosis* (*Mtb*) infection in a hypothetical case pair to illustrate timing of case-pair interval calculations^a^. ^a^The case-pair intervals estimate the duration of latent *Mtb* infection and disease of the secondary patient or the period during which SNPs are expected to accumulate in the sampled *Mtb* genomes for differentiating source and secondary patients' isolates. We calculated the case-pair interval as the observed time difference between specimen collection dates for the source–secondary case pair (T_1_ to T_2_). However, this does not include a portion of the secondary patient's *Mtb* infection before specimen collection for the first patient. To account for this portion, we also calculated a modified case-pair interval, which we defined as the time between the midpoint of the source patient's estimated TB infectious period and the sample collection date of the secondary patient (T_0_ to T_2_).

We aimed to estimate the duration of *Mtb* infection for the secondary patient, representing the time during which SNPs that differentiate the source patient's and secondary patient's isolates are expected to accumulate in the *Mtb* genome. To approximate this period, we calculated a case-pair interval to estimate the time difference between sample collection dates for the source–secondary case pairs in the aggregated cluster data (Figure 2). The case-pair interval is easy to calculate because the sample collection date is almost always available in case reports of culture-confirmed TB and is similar to the duration of *Mtb* infection among secondary patients as long as their infection occurred soon before the source patient's TB diagnosis. However, this interval is a poor proxy of infection duration if infection in the secondary patient occurred closer to the start of the TB infectious period for the source patient. To account for this, we also calculated a modified case-pair interval, which we defined as the time between the midpoint of the source patient's estimated TB infectious period and the sample collection date for the secondary patient (Figure 2). Of note, five case-pair intervals and two modified case-pair intervals were negative, indicating that the secondary patient received a diagnosis before the first patient; we reset these intervals to zero. We could not calculate a case-pair interval for two source cases with missing sample collection dates (i.e., excluded from **Figure 4**). SNP differences and these intervals were characterized by using median and interquartile ranges (IQRs).

We examined the associations between case-pair and modified case-pair intervals and SNP differences between each source–secondary case pair to determine if shorter intervals were linked with smaller pairwise SNP differences. We compared SNP differences between case pairs with modified case-pair intervals of ≤ 2 years, which we defined as recent TB transmission. We assessed multiple mathematical functions to determine which, if any, best fit these data. First, we fit a constant linear function, which assumes that *Mtb* accumulates SNPs at a constant rate throughout latent infection and disease. We also fit a piecewise linear function assuming that the mutation rate during disease might be different from the mutation rate during latent infection. We defined a threshold below which the modified case-pair interval was unlikely to include a long period of latent infection, equivalent to the sum of half the median TB infectious period of included source patients and the median TB infectious period of secondary patients. Secondary patients in pairs with an interval shorter than this period are presumed to have experienced short or no period of latent infection. As such, we assumed their *Mtb* isolates were subject only to the rate of mutation during disease. Secondary patients among pairs with an interval greater than that threshold value likely experienced at least some period of latent infection, and we therefore assumed their *Mtb* isolates were subject to a composite of the mutation rates during disease and latent infection.

This activity was reviewed by CDC and was conducted consistent with applicable federal law and CDC policy^1^; the Los Angeles County Department of Public Health's Institutional Review Board also provided a non-research determination. Analysis code is available at https://github.com/kbratnelson/ltbi-mutation.

## Results

### Aggregated Clusters

**Figure 3:**
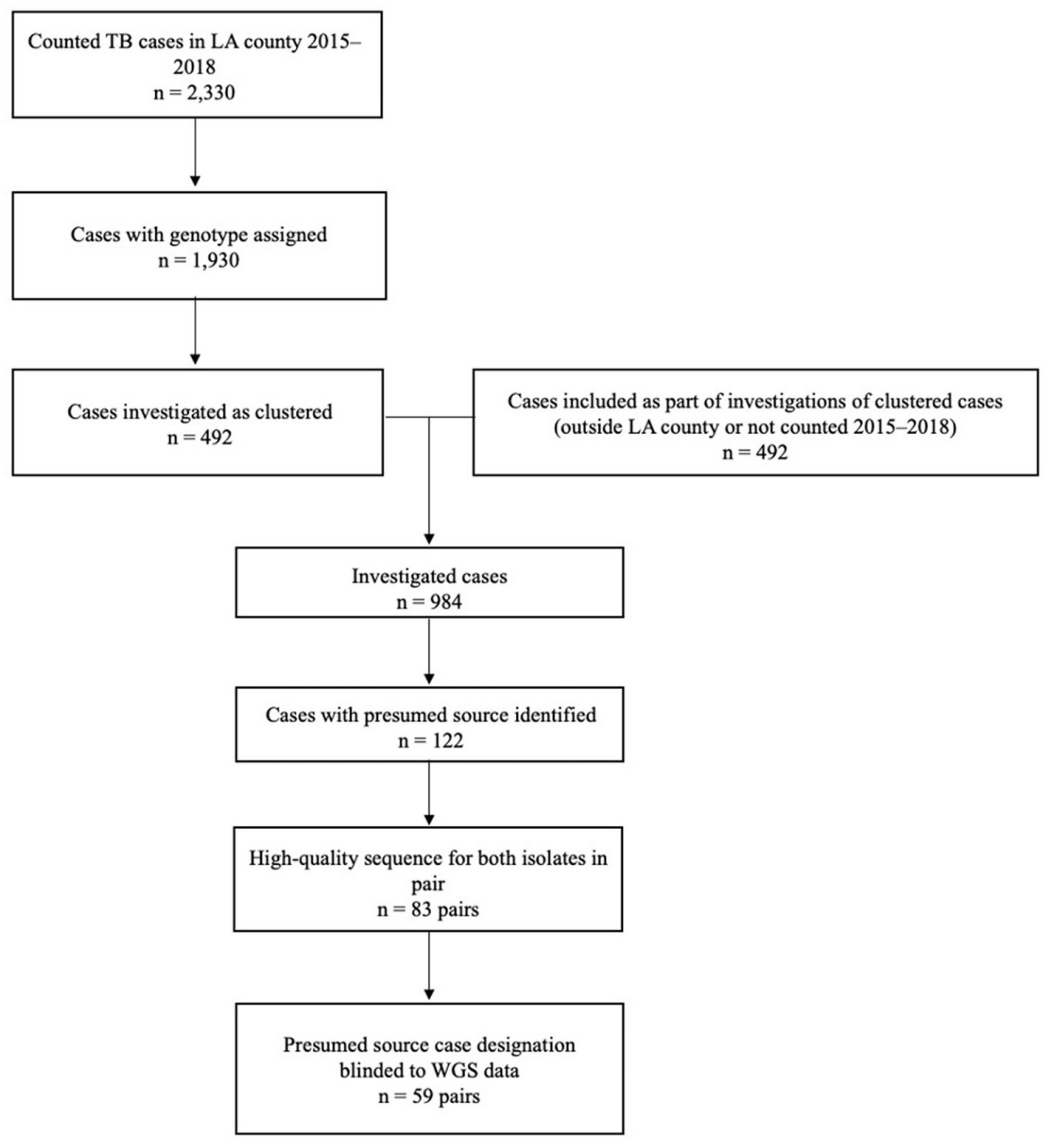
Inclusion criteria for the analytic data set of genotype-matched TB source–secondary case pairs in Los Angeles (LA) County, California, 2015–2018^a^. ^a^Genotype-matched clusters are defined on the basis of a combination of spacer oligonucleotide typing (spoligotyping) and 24-locus mycobacterial interspersed repetitive unit–variable number tandem repeat (MIRU-VNTR) genotyping results. Standardized methods for whole-genome sequencing (WGS) and phylogenetic analysis were applied, as described in the text.

A total of 2,330 TB cases were reported to Los Angeles County Department of Public Health during 2015–2018 (Figure 3). Of these, 83% (*n* = 1,930) were culture-confirmed and assigned a genotype; 492 (25%) of these cases were clustered and further investigated. Molecular surveillance in California and the cluster investigations conducted by Los Angeles County identified an additional 492 cases among patients who had either epidemiologic or genotypic links to the patients identified in Los Angeles County and either resided outside of the county, were reported before 2015 or after 2018, or both. Of the 984 total TB cases investigated, presumed source cases were identified for 12% (*n* = 122). When we restricted analyses to only those cases with high-quality sequences and those for which the results of WGS analysis were not available before the source case was designated (i.e., source case determinations that were not influenced by WGS results), 59 source–secondary case pairs were available for analysis.

One hundred six cases comprised the 59 source–secondary case pairs (9 cases were designated as the presumed source for >1 secondary case and 3 cases were both a secondary case and a presumed source case). These patients were predominantly male (66%; *n* = 62), Hispanic (72%; *n* = 76), and non-US–born (66%; *n* = 70) (Table 1). The majority of cases had been diagnosed in Los Angeles County (97%; *n* = 103). The median size of genotype-matched clusters to which cases belonged was 5 (interquartile range: 3–8). Twenty-six percent (*n* = 28) of patients reported substance use (drug or excess alcohol use), and 7% (*n* = 7) reported experiencing homelessness during the year before TB diagnosis. The proportions of patients with HIV coinfection or other reported potential causes of immunocompromise were low (≤ 5%), except for diabetes mellitus (19%; *n* = 20). Previous TB disease diagnoses were uncommon (2%; *n* = 2). TB patients included in the final analytic data set of source–secondary case pairs were similar to all patients who had undergone investigation, but were on average younger, belonged to smaller clusters, were more likely to be of Hispanic ethnicity, and were less likely to have been homeless during the year before their TB diagnosis (Table 1).

**Table 1:** Demographic, molecular, and clinical characteristics of patients in the analytic data set of genotype-matched TB source–secondary case pairs vs. other investigated cases in Los Angeles County, California, 2015–2018.

**Characteristic**	**Cases included in case pairs** **(*n* = 106) no. (%)**	**Other investigated cases (*n* = 878) no. (%)**
Demographic characteristics		
Sex		
Male	66 (62)	581 (66)
Female	39 (37)	287 (33)
Unknown/missing	1 (1)	10 (1)
Median age (yrs) (IQR)	39 (22–47)	50 (34–63)
Race/ethnicity^a^		
Asian	21 (20)	292 (33)
Black/African American	5 (5)	110 (13)
Hispanic/Latino	76 (72)	428 (49)
White	3 (3)	35 (4)
Unknown/missing	1 (1)	10 (1)
Birthplace^b^		
US-born	35 (33)	250 (28)
Non-US–born	70 (66)	613 (70)
Unknown/missing	1 (1)	15 (2)
TB diagnosis location		
In Los Angeles County	103 (97)	852 (97)
Outside Los Angeles County	3 (3)	26 (3)
Molecular characteristics		
Culture status		
Positive	105 (99)	843 (96)
Negative	0	14 (2)
No results reported	1 (1)	21 (2)
Number of genotype-matched clusters	44	178
Median number of cases (IQR)	5 (3–8)	7 (4–12)
Minimum number of cases	2	2
Maximum number of cases	14	38
Social characteristics		
Any substance use	28 (26)	182 (21)
Excess alcohol use during previous year^c^	16 (57)	137 (75)
Injection drug use during previous year^c^	3 (11)	15 (8)
Non-injection drug use during previous year^c^	17 (61)	86 (47)
No reported history of substance use during previous year	75 (71)	650 (74)
Unknown/missing	3 (3)	46 (5)
Homelessness		
Homeless during the year before diagnosis	7 (7)	120 (14)
No known history of homelessness during the year before diagnosis	98 (92)	744 (85)
Unknown/missing	1 (1)	14 (2)
Incarceration		
TB diagnosed while patient was incarcerated	0	24 (3)
TB diagnosed while patient was not incarcerated	105 (99)	844 (96)
Unknown/missing	1 (1)	10 (1)
Clinical characteristics		
HIV testing		
Positive	1 (1)	30 (3)
Negative	88 (83)	656 (75)
Not offered	7 (7)	66 (8)
Refused	0 (0)	7 (1)
Unknown/missing	10 (9)	119 (14)
Other		
Immunosuppression other than HIV	0	45 (5)
Tumor necrosis factor-α antagonist therapy	0	8 (1)
Post-organ transplantation	0	7 (1)
Diabetes mellitus	20 (19)	231 (26)
End-stage renal disease	1 (1)	26 (3)
Any first line-drug resistance	4 (4)	107 (12)

*IQR, interquartile range*.

a *Hispanic/Latino includes all persons with Hispanic/Latino ethnicity. Other categories include persons with non-Hispanic/Latino ethnicity and the respective race*.

b *US-born is based on eligibility for citizenship at birth and includes people born overseas to parents who are US citizens*.

c *Frequencies of patients with excess alcohol use and injection and non-injection drug use reported during the previous year are tabulated among the subset of patients reporting any substance use. These categories of substance use are not mutually exclusive (i.e., a patient with multiple substance use may be counted multiple times)*.

The epidemiologic links between the 59 source–secondary case pairs were usually classified as definite (93%; *n* = 55) (Table 2). Thirty-seven (63%) pairs were between named contacts; 15 (25%) were linked through a shared location; 6 (10%) were among family members; and 1 (2%) had shared contacts in a social network. The estimated median TB infectious periods of source and secondary patients were 202 and 144 days, respectively.

**Table 2:** Epidemiologic and infectiousness characteristics of genotype-matched TB source–secondary case pairs in Los Angeles County, California, 2015–2018, included in the analytic data set.

**Characteristic**	**Case pairs** ***n* = 59**
Strength of epidemiologic link^a^	
Definite	55 (93)
Probable	4 (7)
Possible	0 (0)
Type of epidemiologic link	
Named contact	37 (63)
Shared location	15 (25)
Family member	6 (10)
Shared contact(s) or social network	1 (2)
Infectiousness of presumed source case	
Pulmonary, smear-positive or cavitary	46 (96)
Pulmonary, smear-negative and non-cavitary	2 (4)
Median TB infectious period of presumed source patients (days) (IQR)	202 (132–287)
Median TB infectious period of secondary patients (days) (IQR)	144 (121–186)

*IQR, Interquartile range*.

a *See Appendix Table 2 for definitions of definite and probable epidemiologic links*.

*Mtb* isolates from the 59 case pairs were predominantly Euro-American lineage (75%) but included East Asian lineage (12%) and Indo-Oceanic lineage (14%) as well. Three case pairs from different genotype-matched clusters had large SNP differences that were inconsistent with a direct transmission event (16, 27, and 58 SNPs for case pairs with modified case-pair intervals of 7.5 months, 1.4 years, and 6.2 years, respectively) and were excluded from the analysis. Among the remaining pairs, pairwise SNP differences ranged from 0 to 10 (median: 1; IQR: 0–2). Case-pair intervals ranged from 0 to 14.5 years (median: 304 days; IQR: 74–935 days), and modified case-pair intervals ranged from 0 to 15.1 years (median: 197 days; IQR: 19–827 days) (Appendix Figure 1). Four case pairs with modified case-pair intervals of ≤ 2 years had isolates >5 SNPs apart. Although these cases had definite epidemiologic links, further inspection of the direction of genetic change indicated that isolates in all 4 of these case pairs diverged in different directions from a hypothetical MRCA. The same was true for 4 of the 5 case pairs with modified case-pair intervals of >2 years and isolates >5 SNPs apart.

**Figure 4:**
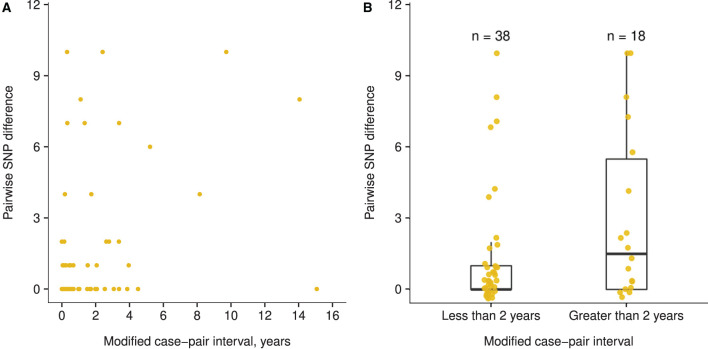
Association between modified case-pair interval and pairwise single nucleotide polymorphism (SNP) difference between genotype-matched source–secondary case pairs in Los Angeles County, California, 2015–2018^a^. ^a^The modified case-pair interval is defined as the time between the estimated midpoint of the source patient's TB infectious period and the sample collection date of the secondary patient. Each dot corresponds to a source–secondary case pair. **(A)** Scatter plot of modified case-pair interval, in years, against pairwise SNP distance. **(B)** Pairwise SNP differences of case pairs with modified case-pair intervals defined as recent transmission (during the previous 2 years) or reactivation (transmission occurred >2 years ago).

The trend between the modified case-pair interval and SNP differences was not linear (Appendix Figure 2A), and a small difference occurred in the pairwise SNP difference between case pairs with a modified case-pair interval of ≤ 2 years (i.e., recent transmission) (median: 0 SNPs; IQR: 0–1 SNPs), and those with an interval of >2 years (median: 1.5 SNPs; IQR: 0–5.5 SNPs) (Figure 4). The functions we fit to these data both showed low *R*^2^ values (≤ 0.10), indicating poor fit of both linear and piecewise linear models (Appendix Figure 2). Based on the distribution of time data, we defined relatively longer modified case-pair intervals as >3 years. Notably, isolates from multiple case pairs with longer modified case-pair intervals (>3 years) were approximately identical to those of the source patients on the basis of sequencing. The trends between case-pair interval and SNP distance were similar to those between modified case-pair interval and SNP distance (data not shown).

### Single-Cluster

**Figure 5:**
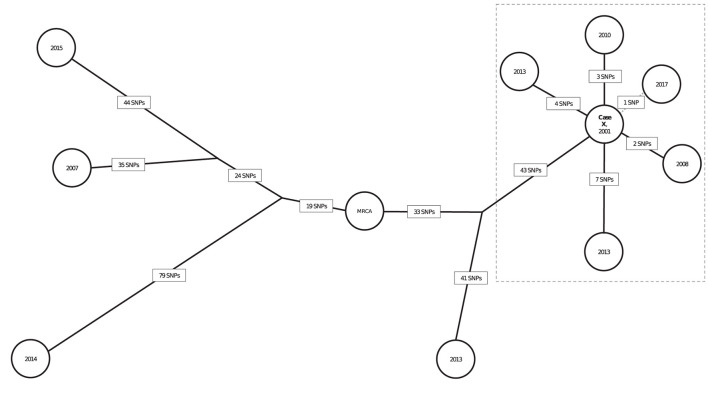
Neighbor-joining tree (phylogenetic analysis) representing the genetic distances in single nucleotide polymorphisms (SNPs) among 10 genotype-matched isolates of *Mycobacterium tuberculosis* (*Mtb*), including isolates from a school-based cluster in California, 2001–2017^a^. ^a^Each node (circle) represents a patient's *Mtb* isolate in the cluster. The year in each node corresponds to the year of TB diagnosis. SNP distances between each isolate are displayed in boxes on each branch. Bootstrap values obtained from 500 replicates were 100% for all branches. The isolates within the dotted box are the most closely related in the cluster and belong to patients for whom epidemiologic links were identified. Branch lengths were scaled proportionally among these isolates to improve visualization. Case X was diagnosed in 2001 and identified as the most likely source for 4 cases and a possible source for the fifth (2017) case in the dotted box; diagnosis dates for these cases ranged from 2008 to 2017. Solid black lines between Case X and other cases indicate that a definite or probable epidemiologic link was identified; the dotted line represents that a possible epidemiologic link (see Appendix Table 2) was identified. Three genotype-matched isolates not shown are from non-US–born patients whose TB was diagnosed elsewhere in the United States, indicating their cases are unlikely related to the cluster of interest.

The SNP distances for the 10 genotype-matched cases in California are illustrated in a phylogenetic tree (Figure 5); 6 isolates are from patients with definite or possible epidemiologic links to a patient with infectious TB in a California school (Case X). Case X was diagnosed in 2001 and is the most likely source for 5 other cases in that cluster. Five presumed secondary cases were diagnosed in 2008 (1 case), 2010 (1 case), 2013 (2 cases), and 2017 (1 case). Four of these patients had clear epidemiologic links to the 2001 source patient established through a 2013 cluster investigation. Duration of latent infection appears to be related to SNP differences; the *Mtb* isolate from the case diagnosed in 2008 had a pairwise difference of 2 SNPs relative to the source patient's isolate; the 2010 isolate was 3 pairwise SNPs from the source patient's isolate; and the isolates from 2013 were 4 and 7 SNPs different from the source patient's isolate, respectively. However, the isolate from the case diagnosed most recently, in 2017, was most closely related genetically to the index Case X (1 SNP). Of note, the possible epidemiologic link between these 2 patients involved a connection to the same neighborhood but no known direct contact, allowing for the possibility that another infectious case in the cluster might have been the source for the 2017 case. The 4 other isolates were from cases diagnosed during the same period but had large SNP differences (range: 84–174 SNPs) and therefore were ruled out as being part of the transmission chain involving Case X.

## Discussion

Our analysis of 59 source–secondary case pairs derived from a diverse set of clustered cases investigated in Los Angeles County did not indicate a linear association between SNP distance and modified case-pair interval, a proxy for duration of latent *Mtb* infection and TB disease. Efforts to fit a piecewise linear function to these data also were unsuccessful, which underscores the lack of a clear association between modified case-pair intervals and SNP difference. Furthermore, the range of pairwise SNP distances observed for cases with modified case-pair intervals >2 years (0–10 SNPs) was the same as that for cases with intervals ≤ 2 years, indicating that SNP distance alone is insufficient for differentiating between cases attributable to recent transmission and cases attributable to reactivation from transmission that occurred during the remote past. Although TB programs might consider cases with isolates that are >5 SNPs apart unlikely to be related by recent transmission, 4 case pairs with definite epidemiologic links and modified case-pair intervals of ≤ 2 years had isolates >5 SNPs apart. However, the direction of genetic change was inconsistent with direct transmission. Examination of the direction of genetic change can provide additional information beyond an absolute SNP distance: isolates that diverge in different directions may be less likely to be related through direct transmission than if the isolate from the secondary case had been directly descended from the isolate from the presumed source case. Separately, we determined that longer presumed infection periods were inconsistently associated with larger SNP differences on the basis of detailed examination of a single, well-characterized cluster of TB cases in California with a nationally uncommon genotype. We observed cases attributable to reactivation of latent infection that were epidemiologically linked to a source case in the remote past and had isolates with few SNP differences relative to this presumed source's isolate. These analyses indicate that no universal rule can be applied consistently to genomic data from a TB cluster to determine the time when specific patients acquired their *Mtb* infection, which implies that WGS alone cannot be used to determine definitively that a case is attributable to recent transmission.

Although a constant rate of mutation during latent infection similar to that during disease might have indicated that cases with longer latent periods would accumulate more mutations than those with shorter latent periods, this is not supported by our data or uniformly by published studies. We reviewed 4 epidemiologic and 2 laboratory (animal model) studies that estimated the mutation rate of *Mtb* during latent infection (Appendix Table 1). Two epidemiologic studies reported a similar mutation rate during latent infection and disease (18, 19), and two others reported a lower rate during latent infection (20, 21). The two laboratory studies reported either a similar mutation rate between latent infection and disease among non-human primates or that *Mtb* replicates throughout the course of chronic infection of mice (22, 23). Considerable uncertainty remains regarding the mutation rate of *Mtb* during latent infection. While robust genomic and epidemiologic data from TB case investigations can provide some insight into the expected number of genetic differences between the *Mtb* isolates of patients linked through transmission, they are limited by the fact that it is difficult to infer the duration of latent infection.

Bacterial WGS has revolutionized molecular characterization of *Mtb* (24). Compared with previous strain-typing methods, which examine <1% of the *Mtb* genome, analyses of WGS data characterize >90% of the genome at the level of individual base-pairs, enabling measurement of fine-scale variation approximately genome-wide. Employing WGS for identifying evidence of recent TB transmission is often more discriminatory than traditional genotyping methods: whereas conventional genotyping can indicate that two isolates are a match on the basis of a small part of the genome, WGS can reveal additional sequence diversity that can be epidemiologically relevant (7). However, despite these technologic advances, this report illustrates why phylogenetic analyses should be interpreted alongside available clinical and epidemiologic information for accurately characterizing clusters of TB cases and guiding further control efforts. Our findings largely support studies that have demonstrated the importance of integrating epidemiologic and genomic data to make inferences about TB transmission events in settings of low TB incidence (13, 25, 26). To facilitate this data integration, DTBE has developed a logically inferred TB transmission algorithm (LITT) (27). A LITT user's manual, training (mock) data sets, training presentation, input file templates, and all code written in R^(28)^ are available at https://github.com/CDCgov/TB_molecular_epidemiology. Our findings support the use of tools such as LITT, which allow for systematic integration and interpretation of genomic and epidemiologic data to guide outbreak investigation in the U.S.

In the United States, investigation of transmission chains is facilitated by a relatively low TB incidence, routine contact investigations, and a well-established molecular surveillance system. National surveillance data indicate that the majority of (>80%) TB cases were attributable to reactivation of infection acquired >2 years prior (remote transmission); while many of these reactivation cases are likely to have resulted from infection acquired outside of the United States, some cases are probably the result of remote transmission within the United States (28, 29). However, local TB programs still spend substantial resources investigating TB clusters that might involve recent transmission. Among certain populations, suspected cases of recent transmission share *Mtb* strains that have persistently circulated in the same area over many years, leading to large clusters of common genotypes with transmission links that are difficult to resolve, even with WGS. WGS can reveal that these clustered isolates are all closely related with few (≤ 5) SNP differences, placing the burden on public health officials to determine through epidemiologic investigation if these similarities are attributable to recent transmission or reactivation of a longstanding latent infection with the same, epidemiologically entrenched strain. In these cases, resource allocations for investigation might need to be prioritized. For example, referring back to contact investigation records of past TB cases might identify previously unrecognized epidemiologic links or a contact who was not fully evaluated or treated to prevent progression to disease, allowing the TB program to conclude a recent TB case is attributable to reactivation of latent infection. This review process can be challenging and time-consuming if records are incomplete or unavailable. Conversely, occurrence of multiple cases in a short timespan might indicate recent transmission rather than concomitant reactivations of TB disease. In that scenario, contact investigations for current cases might be expanded, or cluster investigations to identify epidemiologic links among recent cases might be initiated. WGS data can serve to guide these decisions, but our analysis reveals that an overreliance on these data might not serve prevention efforts well. Thus, a better understanding of the expected rate of genetic change during latent *Mtb* infection can help guide public health practitioners in allocating resources more effectively.

Our study has multiple limitations. First, epidemiologic studies that aim to understand mutation rates during *Mtb* infection are inherently limited by the inability to sample *Mtb* from latently infected persons. We used the genetic profile of *Mtb* from the source case as a proxy for the sequence upon infection of the secondary case. In other words, we assumed that the source *Mtb* sequence did not change and all mutations occurred in the secondary case. This assumption may be plausible given that we generally observed that *Mtb* isolates from presumed source cases did not have mutations that were absent from the secondary cases' isolates. However, it is unclear to what extent *Mtb* bacteria present in sputum sampled for genotyping accurately represent either the clonal population of bacteria in the lungs or the genetic makeup of bacteria transmitted to secondary cases. Advanced tools for detecting minor genomic variants, including deep sequencing techniques, might prove important in understanding the clonal variation present during infection and disease, which can be useful for defining transmission events (30–32). Because this is typically unavailable at a programmatic level, our analysis focuses on interpretation of WGS as implemented in the United States. Second, source–secondary case pairs might have been identified incorrectly or the case-pair intervals we calculated might be inaccurate. Source–secondary case pairs were identified by local public health as the most likely transmission pathway on the basis of available data; however, these links might not be correct especially if the true source case was undiagnosed or not genotyped. Cluster investigation practices were developing during 2015–2018, and resources applied to cluster investigations were inconsistent. Possibly, the investigators identified fewer case pairs during the initial years, which might have affected the completeness of the source–secondary case-pair data. Also, source determinations based on epidemiologic links are more likely to be made with cooperative patients and both epidemiologic and transmission links may be more frequently established for contacts identified in the recent past (e.g., recall bias). We used the modified case-pair interval between source–secondary case pairs as a proxy for the duration of latent infection of the secondary case. Diagnostic delays, estimated in a cohort study of 158 patients in Maryland to be >90 days for approximately half of TB cases, can lead to this period overestimating the duration of latent infection if the secondary patient in a pair did not seek care soon after progressing to TB disease (33). This can affect our results if diagnostic delays are attributable to pathologies that also affect disease progression (e.g., HIV infection status). Such factors have been reported to influence mutation rates during disease and likewise might influence mutation rates during latent infection (34). However, few HIV-positive cases existed in the clusters we analyzed, and any stratification by case characteristics was limited by our sample size. Third, the single-cluster investigation involving the school that we studied spanned 2001–2017; although genotyping of the presumed source case diagnosed in 2001 was performed retrospectively, universal genotyping of all TB cases in the United States with spoligotyping and 24-locus MIRU-VNTR did not begin until 2009. Thus, cases in this cluster that were involved in transmission might not have been genotyped and therefore not investigated. Fourth, accurately identifying presumed source cases among TB patients with nationally common genotypes is difficult, given the relatively larger number of potential source cases. Consequently, the source–secondary pairs in our data are biased toward rarer genotypes, and mutation rates are known to vary by genotype. Fifth, we simplify the stages of *Mtb* infection into two discrete states, latent infection and TB disease. We acknowledge that this progression is better represented as a spectrum of disease, across which mutation rates can gradually change; this idea is supported by recent work that reviewed the epidemiologic evidence for discrete disease states and reported it to be lacking (35). Lastly, our results are not necessarily representative nationally given the focus on a single county where investigated cases had presumed source cases identified and WGS data were available but blinded to avoid biasing source case designations.

Implementation of routine WGS for TB cases in the United States will continue to provide more data for answering questions regarding how to combine and interpret epidemiologic and genomic data for guiding TB transmission control. Methods for integrating clinical and epidemiologic data with surveillance databases and phylogenetic analysis results can guide conclusions regarding both the likelihood of recent transmission and the likely directionality, settings, and social drivers of that transmission. Building such systems should be a future priority for providing local public health practitioners with better tools for monitoring and investigating TB transmission. The ability to more accurately interpret WGS in the context of other data sources can lead to a clearer understanding of TB epidemiology and ultimately improve TB prevention measures in such low-incidence settings as the United States.

## Author's Note

The findings and conclusions in this article are those of the authors and do not necessarily represent the views, opinions, or official position of the Centers for Disease Control and Prevention, the California Department of Public Health or the California Health and Human Services Agency.

## Data Availability Statement

The data analyzed in this study are subject to the following licenses/restrictions: Data were shared for the purposes of this project under a data use agreement and are only available *via* state and local public health department jurisdictions due to necessary patient privacy protections. Requests to access these datasets should be directed to Sarah Talarico, mzi4@cdc.gov.

## Ethics Statement

The studies involving human participants were reviewed and approved by Centers for Disease Control and Prevention Institutional Review Board and Los Angeles County Department of Public Health's Institutional Review Board. Written informed consent for participation was not provided by the participants' legal guardians/next of kin because this data was collected as a part of standard public health activities. Written informed consent was not obtained from the individual(s) for the publication of any potentially identifiable images or data included in this article.

## Author Contributions

KN: conceptualization, formal analysis, writing—original draft, and writing—review and editing. ST and CM: conceptualization, formal analysis, data curation, writing—original draft, and writing—review and editing. SP, MC, AC, WN, and TS: investigation, writing—review and editing. KR, LC, and JP: data curation and writing—review and editing. BS: conceptualization, writing—original draft, and writing—review and editing. All authors contributed to the article and approved the submitted version.

## Conflict of Interest

The authors declare that the research was conducted in the absence of any commercial or financial relationships that could be construed as a potential conflict ofinterest.

## Publisher's Note

All claims expressed in this article are solely those of the authors and do not necessarily represent those of their affiliated organizations, or those of the publisher, the editors and the reviewers. Any product that may be evaluated in this article, or claim that may be made by its manufacturer, is not guaranteed or endorsed by the publisher.

## Footnotes

^1^See e.g., 45 C.F.R. part 46, 21 C.F.R. part 56; 42 U.S.C. §241(d); 5 U.S.C. §552a; 44 U.S.C. §3501 et seq.
