# Supplementary material for: Mutation of Mycobacterium tuberculosis and Implications for Using Whole-Genome Sequencing for Investigating Recent Tuberculosis Transmission
Source: Front Public Health. 2022 Jan 13;9:790544. doi: 10.3389/fpubh.2021.790544 (PMC8793027; doi:10.3389/fpubh.2021.790544)
Supplement: Supplementary file 1 [file Data_Sheet_1.docx]

**Appendix Table 1**

Selective summary of published epidemiologic and laboratory studies estimating the mutation rate of *Mycobacterium tuberculosis* (*Mtb*) during latent infection.

| **Author** | **Year published** | **Study type** | **Study design** | **Sample size** | **Molecular characterization method** | **Key findings** | **Conclusion, *Mtb* mutation rate during latent infection** |
| --- | --- | --- | --- | --- | --- | --- | --- |
| Lillebaek et al.[19] | 2016 | Epidemiologic | Studied 6 paired *Mtb* clinical isolates, with each pair representing reactivation of LTBI >3 decades after primary infection. | 6 case pairs | WGS | Estimated mutation rate 0.2–0.3 SNPs/year over 33 years, indicating that latent *Mtb* accumulates mutations at rates similar to observations from cases of active disease. | Similar between latent infection and disease |
| Yang et al.[20] | 2011 | Epidemiologic | Studied 13 paired *Mtb* clinical isolates, with each pair representing reactivation of LTBI >3 decades after primary infection. | 13 case pairs | SSRs and PE/PPE region | One pair indicated an insertion in one SSR. In the genome-wide analysis, one pair indicated a 9 base-pair insertion and single base-pair change in the PE_PGRS33 gene, which indicates that LTBI has a low mutation rate during latent infection (i.e., low replication). | Lower during latent infection than disease |
| Ford et al.[23] | 2011 | Laboratory, non-human primate model | Compared accumulation of mutations in *Mtb* during active, latent, and reactivated disease and calculated the mutation rates for each state. | 9 macaques | WGS (Illumina) | *Mtb* acquires a similar number of chromosomal mutations during latency as during active disease. Identity of mutations indicates they are largely attributable to oxidative DNA damage. | Similar between latent infection and disease |
| Colangeli et al.[21] | 2014 | Epidemiologic | Used epidemiologic information to identify 4 secondary cases with the same index case: 2 experienced disease rapidly; 2 after 20 years of latent infection. | 4 case pairs | WGS | Replication rates or mutation rates are lower during latent disease, compared with the 2-year period leading to active TB. No evidence for a higher rate of oxidative damage-induced mutagenesis. | Lower during latent infection than disease |
| Gill et al.[22] | 2009 | Laboratory, mouse model | Measured replication rate by exploiting an unstable plasmid that is lost at a steady, quantifiable rate from dividing cells. Applied a mathematical model to calculate replication and death rates during infection. | 15 mice | NA | *Mtb* replicates throughout the course of chronic infection of mice. | Did not compare |
| Lillebaek et al.[18] | 2003 | Epidemiologic | Studied paired *Mtb* isolates, with each pair representing reactivation of LTBI >3 decades after primary infection. | 201 case pairs | IS6110 RFLP | 14 DNA patterns identified among historical strains were identical to patterns identified among the “recent strains.” The half-life of IS6110 DNA patterns during latency estimated at 36 years (95% confidence interval: 25–54 years). | Similar between latent infection and disease |

**Abbreviations:** LTBI, latent tuberculosis infection; NA, not applicable; RFLP, restriction fragment length polymorphism; SNP, single-nucleotide polymorphism; SSRs, simple sequence repeats; WGS, whole-genome sequencing.

**Appendix Table 2**

Detailed definitions of strengths of epidemiologic links used in the cluster investigation.

| **Definite epidemiologic link** |
| --- |
| 1. Named contact of a patient during the TB infectious period, or  2. Two patients resided in the same location during one patient’s TB infectious period. |
| **Probable epidemiologic link** |
| 1. Two patients were in the same place around the time of one of the patient’s TB infectious period; however, the timing of when the 2 patients resided in the same location or the timing of the infectious period was not certain enough for meeting the definite epidemiologic link criteria, or  2. Two patients share a common contact without naming each other as direct contacts. |
| **Possible epidemiologic link** |
| 1. Two patients lived or worked in the same neighborhood during the same general period, or  2. Two patients shared activities or social or behavioral traits that increased the chances they were in contact with each other, or  3. The two patients are connected on a social network. |

**Appendix Fig. 1.** Distribution of case-pair intervals and modified case-pair intervals between genotype-matched source–secondary case pairs in Los Angeles County, California, 2015–2018^a^.

**
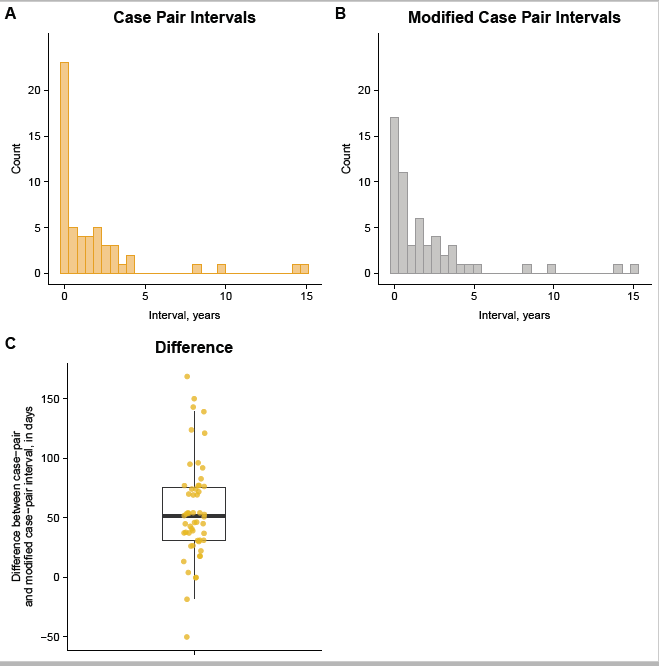
**

^a^ We defined both case-pair and modified case-pair intervals for source–secondary case pairs. A. Distribution of case-pair intervals, or the time between the sample collection date of the source and the secondary patients. B. Distribution of modified case-pair interval or the time between the estimated midpoint of the source patient’s TB infectious period and the sample collection date of the secondary patient. C. Distribution of the differences between modified case-pair intervals and case-pair intervals, in days. Positive differences indicate a larger modified case-pair interval than case-pair interval; negative differences indicate a larger case-pair interval than modified case-pair interval.

**Appendix Fig. 2.** Mathematical functions for assessing best-fit models for characterizing mutation rates during latent *Mycobacterium tuberculosis* (*Mtb*) infection by comparing modified case-pair intervals and pairwise single-nucleotide-polymorphism (SNP) difference between genotype-matched source–secondary case pairs in Los Angeles County, California, 2015–2018^a^.****

^a^ Modified case-pair interval is defined as the time between the estimated midpoint of the source patient’s TB infectious period and the sputum sample collection date of the secondary patient. We assessed multiple mathematical functions to determine which, if any, provided a good fit to the data. A. A constant linear function fit, which assumes that *Mtb* accumulates SNPs at a constant rate throughout latent infection and disease. B. Piecewise linear function fit, which assumes that the mutation rate during active disease might be different from the mutation rate during latent infection. We defined a threshold below which the modified case-pair interval was unlikely to include a long period of latent infection, equivalent to the sum of half the median infectious period of included source patients and the median TB infectious period of secondary patients. Among the pairs in the analytic data set, we calculated this threshold to be 274 days, or approximately 9 months. The secondary cases in case pairs with modified case-pair intervals shorter than this period are presumed to have experienced short or no period of latent infection, and their *Mtb* is therefore subject only to the rate of mutation during disease. Case pairs with a modified case-pair interval greater than this value are subject to a different rate, which is a composite of the rates during disease and latent infection.
